# Using Concept Mapping to Define Indigenous Housing First in Hamilton, Ontario

**DOI:** 10.3390/ijerph191912374

**Published:** 2022-09-28

**Authors:** Michelle Firestone, Nishan Zewge-Abubaker, Christina Salmon, Constance McKnight, Stephen W. Hwang

**Affiliations:** 1 MAP Centre for Urban Health Solutions, Li Ka Shing Knowledge Institute, St. Michael’s Hospital, 30 Bond St., Toronto, ON M5B1W8, Canada; 2 Knowledge Translation Program, Li Ka Shing Knowledge Institute, St. Michael’s Hospital, 30 Bond St., Toronto, ON M5B1W8, Canada; 3 De dwa da dehs nye>s Aboriginal Health Centre, 678 Main St E, Hamilton, ON L8M 1K2, Canada

**Keywords:** participatory research, mixed methods, concept mapping, evaluation, indigenous health, housing first, homelessness

## Abstract

Background: The lasting impact of colonization contributes to the disproportionate rates of homelessness experienced by Indigenous people in Canada. Methods: This study used participatory mixed methods to evaluate an urban, Indigenous-led Housing First program in Ontario to fill knowledge gaps on wise practices addressing the unique dimensions of Indigenous homelessness. Using concept mapping, staff perspectives were engaged to generate 65 unique statements describing program delivery and their interrelationships using a six-cluster map. Results: ‘Team’s Professional Skills’ and ‘Spiritual Practices’ rated high in importance (*mean* = 4.75 and 4.73, respectively), and feasibility (*mean* = 4.31 and 4.33, respectively). While fairly important, ‘Partnerships and Agency Supports’ was ranked least feasible (*mean* = 3.89). On average, clusters rated higher in importance than feasibility. Conclusion: Concept mapping draws from local knowledge, elicits strong engagement, and captured the holistic and client-centred approach of an Indigenous Housing First Model.

## 1. Introduction 

At least 72% of the Indigenous population in Ontario live in urban areas [1]. Research has shown that 1 in 15 Indigenous people in urban centres experience homelessness compared to only 1 in 128 for the general population [2]. In Hamilton, Ontario, about 3% of the city’s total population of 747,000 is Indigenous, yet almost half of all homeless individuals identify as Indigenous [3]. For Indigenous people experiencing homelessness, colonization has played a central role in the housing barriers they face. Housing barriers include poverty, lack of access to culturally appropriate social services and housing, discrimination, mental health problems, and intergenerational trauma resulting from experiences with residential schools and the child welfare system. Systemic racism affects access to housing and supports for Indigenous peoples [4,5]. 

Policies and programs to address homelessness at the provincial and federal level in Canada have shifted dramatically towards the Housing First model in recent years, which provides people experiencing homelessness with immediate access to permanent housing and supports [6]. These changes happened shortly after the At Home/Chez Soi research demonstration project, which showed the effectiveness of the Housing First model in ending chronic homelessness for people with mental illness [7]. In light of these findings, the federal government of Canada announced in 2013 that its Homelessness Partnering Strategy would refocus on the implementation of the Housing First approach [8]. As Housing First programs are implemented and scaled up across Ontario, further research is still needed to fill important knowledge gaps and guide program and policy decisions, particularly in relation to experiences of Indigenous homelessness. A specific definition of Indigenous homelessness that has been established by scholars and community members acknowledges the unique experiences and structural factors Indigenous communities face in accessing homes and housing [9]. This definition recognizes how centuries of colonialism have undermined Indigenous wholistic concepts of home and access to secure housing. Defining Indigenous homelessness also reflects the importance of reaching “grounded emplacement” through housing programs that resist the political and economic conditions that uproot Indigenous life and wellbeing [9]. 

Housing Services (previously called Homeward Bound) is a housing support program that assists Indigenous men, women, and families experiencing homelessness in finding and maintaining housing. This program was launched by De dwa da dehs nye>s Aboriginal Health Centre (DAHAC) in 2015 and follows a Housing First approach. Housing Services provides street and peer outreach services, cultural supports, and wellness and community resource referrals. Housing Services aims to provide direct access to sustainable, safe and affordable housing for Indigenous community by working with closely with community partners in Hamilton. In March 2022, during the COVID-19 pandemic services moved from DAHAC to the Hamilton Regional Indian Centre

There has been little research focused on describing and evaluating wise practices in Indigenous-led programs that meet the needs of Indigenous people who are experiencing homelessness, as well as a lack of funding for Indigenous-specific services and supports in housing [10]. The goal of this research was to identify the unique components of an Indigenous-led Housing First program model and generate possible indicators of successful client housing experiences from the perspectives of program staff. To accomplish this goal, we used Concept Mapping. Concept Mapping is an evidence-based tool that has been successfully implemented in urban First Nations, Inuit and Métis research contexts [11] as it builds on mapping traditions, upholds collective values and opinions, and it requires strong community engagement and participation. This research was co-led by scientists at MAP Centre for Urban Health Solutions at St. Michael’s Hospital and DAHAC. 

## 2. Methods

### 2.1. Research Approach and Governance

This research was nested in a larger study called Walking our Journey: a Collaborative Evaluation of an Indigenous Housing First Program. The main objectives of our larger project were to document the unique Indigenous values and principles of the Housing Services program model and to evaluate their influence on client housing experiences. The evaluation was grounded in local community engagement, upholding First Nations ethical standards such as Ownership, Control, Access and Possession (OCAP^®^). A Research Agreement between MAP, Well Living House and DAHAC was generated to ensure relevance, respect, and representation in our protocols and processes. This ensured every stage of our project was shared by all decision-makers in our project governance. Ethics approval was provided by the DAHAC Board and the Research Ethics Board of St. Michael’s Hospital, Unity Health.

### 2.2. Recruitment 

All Housing Services staff members were invited via email to participate in the study. Participants attended three concept mapping activities: (1) brainstorming, (2) sorting and rating, and (3) map interpretation or diagramming at the De dwa da dehs nye>s location in Hamilton. Participants were asked to complete a brief demographic survey before the sorting and rating session began. The survey was used to gather information about the De dwa da dehs nye>s staff participating in the concept mapping sessions.

### 2.3. Concept Mapping Activities

Concept mapping was selected as a research tool that supports local knowledge and establishes a conceptual foundation upon which data measurements and systems can be grounded [11,12]. Concept mapping is a very inclusive process that requires input from participants at each stage, it uses the language of the participants rather than terms imposed by an evaluator, researcher or planner and it generates a graphic representation which at a glance shows all of the major ideas and interrelationships [11,12, 13]. Concept mapping integrates several qualitative and quantitative methods into a series of structured steps. Each participant must complete three data gathering activities: (1) brainstorming, (2) sorting and rating, (3) and map interpretation or diagramming. Concept mapping activities were conducted in Hamilton at DAHAC between April and May of 2019.

#### 2.3.1. Brainstorming 

A 90-min brainstorming session was conducted by the research team. A facilitator asked participants to verbally provide statements in response to the following focal prompt:

*“Delivering Housing Services involves the following component or service…”*

135 statements were generated by the participants which were narrowed down through the statement synthesis process. Four members of the research team met twice to conduct this extensive process to review each statement in detail. Duplicate statements were removed, similar statements were combined and statements that did not answer the focal prompt were removed. The final statement list comprised of 65 statements.

#### 2.3.2. Sorting and Rating 

A two-hour sorting and rating session was conducted by the research team. The final list of 65 statements derived from the statement synthesis process was given to the participants. The facilitator reminded the participants of the focal prompt (the previous brainstorming activity) and then reviewed the statement synthesis process and the final list of 65 statements. They were then asked to complete two activities. First, participants were asked to work independently to sort the statements into groups (themes) that made sense to them and to name each group they created. Next, participants were asked to rate each group of statements on a Likert scale of 1–5 based on the group’s importance to having a positive Housing Services program experience and the group’s feasibility of implementation into Housing Services. 

Each participant was given a unique, non-identifiable code that linked their data to their demographic information. Participants’ data were entered into the Concept Systems^®^—CS Global MAX software which conducted two statistical analyses—multi dimensional scaling and hierarchical cluster analysis—to display the maps in pictorial form for participant analysis in the mapping session. All participant input is measured equally through the multidimensional scaling and hierarchical cluster analysis. Multidimensional scaling combines the data collected in the sorting stage to create a point map. Each point on the map represents one of the statements from the final statement list. The closer the point is to another indicates they were sorted together by the majority of participants. Hierarchical cluster analysis is used to identify the clusters in the map through drawing boundaries around similarly sorted points. 

#### 2.3.3. Map Interpretation Session 

A two-hour mapping session was conducted by the research team. A point map was shown to the participants first where each point represented one of the final 65 statements. Next, a cluster map was shown to the participants that displayed boundaries around the points to show each cluster’s reflection of how the majority of participants sorted the points into distinct groups during the sorting and rating sessions. The participants were instructed to analyze the cluster map, move any points that did not belong in a cluster into another, and provide a unique label for each cluster. Once the map was finalized, the group was then shown their importance and feasibility cluster rating maps based on how they rated each statement. The research team conducted further data analyses separately using the group specific final cluster and rating maps to create Go Zones and pattern matches. A Go Zone is a bivariate graph of the statement values comparing two variables and is divided into four quadrants based on statements rated above average and statements rated below average. The Go Zone displays areas of agreement and differences between two ratings, participants groups, etc. Pattern matching is a method that produces a ladder graph that compares the average cluster ratings for a single variable by single or multiple groups, incorporating data from the demographic questions. 

## 3. Results 

### 3.1. Sample Characteristics

Out of 14 total Housing Services program staff, 13 unique individuals participated in at least one concept mapping activity. This included 12 participants for brainstorming, 11 for sorting and rating, and 9 for the mapping session. Of those in the mapping session, eight participants attended all three sessions and one participant attended one session. During the brainstorming session, participants completed a brief sociodemographic questionnaire. As Housing Services had been established for 4 years, staff employment ranged from 5 months to 4 years, with a mean of 1.9 years. Staff had worked in the homelessness sector and Indigenous organizations for mean 4.7 years and 5.2 years, respectively. Half of the participants identified as Indigenous and the majority were between the ages of 25 and 44. 

### 3.2. Cluster Map

**Figure 1:**
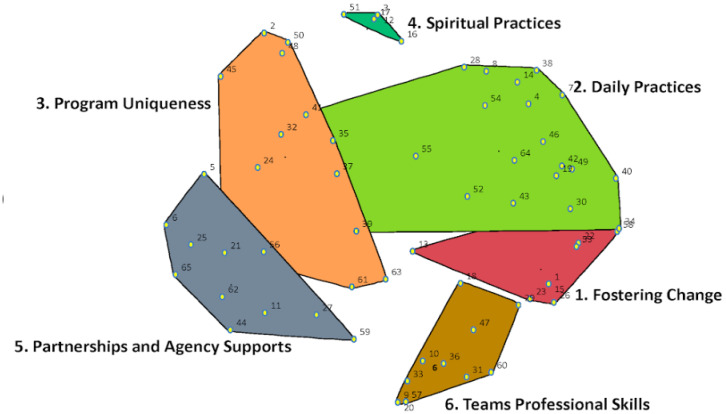
Cluster Map.

During the map interpretation session, the research team presented a seven-cluster and six-cluster computer-generated map to participants. Through an engaging discussion and review of cluster content, participants reached consensus that a six-cluster map was the best fit for the 65 statements. The stress value of the six-cluster map was 0.31. Stress value is a statistic that indicates the goodness of fit of the concept map. A low stress value indicates a better fit and the 0.31 of the six-cluster final map suggests a good fit. Trochim has noted an acceptable range for a stress value for a concept map to be between 0.205 and 0.365 [14]. The six clusters were labeled by participants as: 1. Fostering Change, 2. Daily Practices, 3. Program Uniqueness, 4. Cultural Practices, 5. Partnership and 6. Agency Supports and Team’s Professional Skills. A description of each cluster and the final map can be found below (Figure 1 and Table 1).

#### 3.2.1. Fostering Change

Participants articulated how the Housing Services program model supports clients with their needs in the Fostering Change cluster. The program ensures that clients’ basic needs are met in terms of housing, food, clothing, and crisis support. Once clients are housed, staff can work on strengthening community around the clients to foster change. Examples of statements in this cluster included: ‘working on client goals at their own pace’, ‘staff help clients to develop a toolbox with coping strategies, support and resources’, and ‘staff supporting clients self-image and growth once housed.’

#### 3.2.2. Daily Practices

This cluster provides a comprehensive overview of the activities and services offered through the program. The statements in this cluster reflect the everyday practices and roles of staff on the Housing Services team. From referrals, to case-planning and special events, the Daily Practices cluster describes the activities performed on a regular, ongoing basis for clients. Statements included: ‘staff provide home healthcare visits’, staff accompany clients to appointments’, ‘program provides housing allotments to clients’, staff addressing food insecurity for clients’, and ‘program providing harm reduction supplies and supports.’ 

#### 3.2.3. Program Uniqueness

Participants shared how the Housing Services program works differently from mainstream Housing First programs by meeting the specific needs of the Indigenous community. The Program Uniqueness cluster consists of statements that highlight program activities and processes that align with and uphold Indigenous ways of knowing and doing. Examples of statements in this cluster included: ‘During intake, not relying on acuity and focusing on stories’, ‘Re-establishing connection to Indigenous community for clients’, ‘providing health services and education in community settings’, ‘program accepts referrals from all sources, family, community and By-Name list’, and ‘Program offering different housing options (e.g., rooming house pilot).’

#### 3.2.4. Spiritual Practices

The statements within the Spiritual Practices cluster describe specific program elements such as access to ceremonial and traditional teachings and protocols. Statements included: ‘Program provides access to traditional culture for clients (e.g., providing teachings), ‘program re-established connection to Indigenous community for clients’, ‘Staff provide smudging in clients’ homes’, ‘Program housed within an agency that offers services from waters of life to returning to the spirit world’, and ‘Program housed within organization that has traditional healers.’

#### 3.2.5. Partnership and Agency Supports

Participants conceptualized this cluster around the types of relationships that are developed and required within the Housing Services Program. Statements in this cluster referred to relationships within the program itself, within the larger Aboriginal Health Access Centre, and across other agencies and stakeholders such as landlords and Ontario Aboriginal Housing. Partnerships that are built on trust allow for flexibility in response to community needs and have clear boundaries with respect to the roles and responsibilities that are critical to the Housing Services Program. Examples of statements were: ‘Staff build and manage trusting landlord relationships’, ‘Program has support from all levels of DAHAC management to address bureaucratic barriers’, ‘Team lead and senior management understanding and supporting all staff roles’, and ‘Program partners with Ontario Aboriginal Housing for supportive housing.’

#### 3.2.6. Team’s Professional Skills

Participants grouped statements that described the values and priorities of program staff into the Team’s Professional Skills cluster. This included ways in which the staff supports one another and looks after their own wellbeing in addition to centering client needs and expectations within reasonable boundaries. Statements from this cluster included: ‘Staff recognizing and drawing boundaries with clients’, ‘Program consists of a supportive, close and trusting team’, ‘Program’s approach is inclusive of clients’ full identities’, ‘Staff engage in self-care’, ‘Staff self-reflecting on their biases in client relationships’, and ‘Managing and setting client expectations of program at intake.’

### 3.3. Cluster and Statement Ratings

The Team’s Professional Skills were rated as the most important cluster (mean = 4.75), and the third most feasible group to act on (mean = 4.31). The second most important cluster (mean = 4.73) of Spiritual Practices, as rated by participants, was also rated as the second most realistic cluster to implement (mean = 4.33). Partnerships and Agency Supports were identified as the third most important cluster (mean = 4.56), yet was ranked as the least feasible cluster of statements (mean = 3.89). Program Uniqueness was listed as the fourth important cluster and the second least feasible (mean = 4.55, 4.14). Daily Practices were the second least important cluster, and the fourth feasible cluster (mean = 4.35, 4.18). Finally, Fostering Change was rated the least important cluster (mean = 4.47), despite being rated as the most feasible cluster (mean = 4.36). As noted above, the ratings across the five clusters were high, however there are differences between the relative ratings of importance and feasibility by cluster to explore in the pattern matches and individual statements. The clusters had a mean rating of 4.57 for importance, and 3.88 for feasibility. Table 1 outlines the individual statements organized by each cluster. Each statement is sorted into a ‘high’ or ‘low’ category based on whether their mean ranked value was more or less than the mean for all ranked statements for importance or feasibility. For example, statement 2 has a mean importance ranking of 3.5, making it lower than the overall mean importance ranking of 4.57, meaning it is listed as ‘low’ on Table 1.

### 3.4. Pattern Matches 

**Figure 2:**
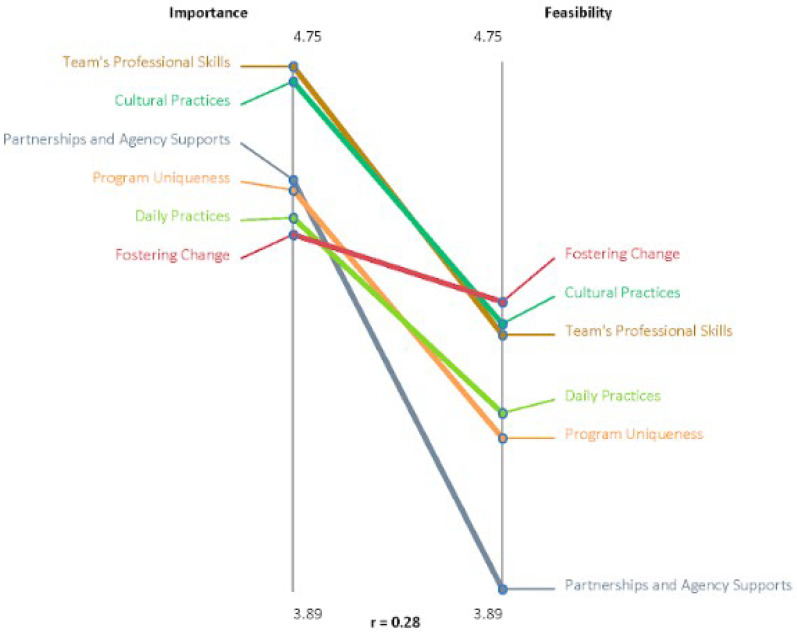
Pattern match, importance vs. feasibility.

We further explored rating data through pattern matching. This analysis compares the average ratings of the clusters between the two rating questions on a graph. Figure 2 outlines a visual representation of the pattern match. There was a low correlation (r = 0.28) between the mean ratings for importance and feasibility, indicating there are important differences between the two ratings to parse out. Multiple pattern matches were generated to identify any within-group differences based on Indigenous identity, or length of experience with Housing Services, Indigenous organizations, and/or the homelessness sector. Notably, the clusters were consistently rated across all groups, with no statistically significant differences between them (*p* < 0.005). Consistently, all concept clusters were rated with a higher mean for importance than for feasibility. This was most strongly demonstrated by the fostering change cluster, where the average importance mean was ranked the lowest at 4.47, and the average feasibility mean the highest at 4.36. Multiple other clusters did not experience relatively significant changes in ranking from importance to feasibility or relative mean. This included the team’s professional skills and cultural practices clusters. The cluster with the largest change in mean ranking was the partnership and agency supports (importance mean = 4.56, feasibility mean = 3.89). When a *t*-test was run comparing the cluster rankings for importance and feasibility, the means were statistically significant (*p* < 0.005). While this was rated the third most important cluster, it was rated significantly lower for feasibility. 

### 3.5. Go Zone Analysis

**Figure 3:**
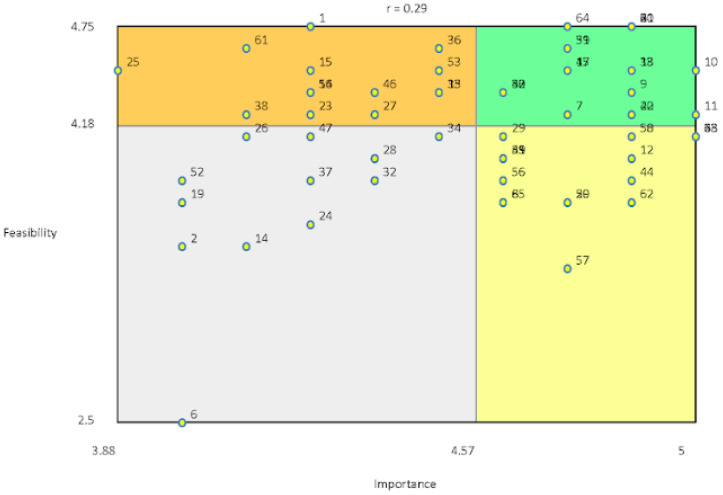
Go Zone.

The Go Zone diagram represented in Figure 3 shows how participants rated a statement’s importance for a positive Housing Services experience and how feasible they would be to implement. The upper right-hand quadrant of the Go Zone contains the statements that were rated most important and most feasible. The statements in this quadrant describe the holistic and strength-based approach of the program and the trusting, accountable, and positive roles played by staff. Statements that were rated as least important and least feasible appeared in the lower left-hand quadrant. These included statements such as *‘opening services to broader Indigenous community’, ‘program partners with Ontario Aboriginal Housing for supportive housing’ and ‘staff accompanying clients to appointments (hospital, social services).’*

## 4. Discussion 

Concept mapping methods were used to elicit the program components and approaches of a unique Indigenous Housing First model in Hamilton, Ontario. Concept mapping aligns with Indigenous community processes and governance as an inclusive, participatory method that facilitates shared decision making and ownership at each stage [11,12]. Participants generated 65 unique statements that reflect the delivery of key components and services of the Housing Services Program. Through a lively and rich dialogue, participants reached consensus on a final map that contained six clusters that visually represented a unique Indigenous Housing First model. Participants identified program elements across client, staff, organizational, community and spiritual levels which collectively contribute to the housing journey of Indigenous people experiencing homelessness in Hamilton. 

Of the six clusters on the final map, participants rated the Spiritual Practices and Team’s Professional Skills as both the most important and most feasible compared to the other clusters. Evidence indicates that health and social programs and services in urban contexts that are grounded in Indigenous practices and ways of knowing and doing result in sustainable impacts that improve overall health and wellbeing for Indigenous people [15,16,17]. Housing Services is a culturally safe program that builds on and strengthens existing social networks and takes a holistic approach to supporting Indigenous people experiencing homelessness that includes mind, body and spirit. Culture has been shown to play an important role in healing for Indigenous communities and is featured throughout recent definitions of Indigenous Homelessness [9]. 

Central to the roles and responsibilities of staff at Housing Services is the way they work with their clients and honour them as full human beings. The Housing Services program aims to walk alongside their clients on their journey from the street to safe and appropriate housing of their choice. This work is realized through internal, organizational supports, and team building as prioritized by DAHAC. As reported elsewhere, housing program success stems from a foundation that centres around a localized understanding of the Indigenous population, their stories and strengths [18]. As illustrated by the cluster map (Figure 1), Partnerships and Agency Supports was rated as the least feasible by participants; specifically around managing partnerships with external groups and stakeholders. This is not surprising given the current Canadian context around lack of affordable and safe housing and siloed funding structures at Federal and Provincial levels that hinder local service integration. Urban Indigenous-led housing and social services operate under the continued imposition of colonial systems and without recognition or funding for the “For Indigenous, By Indigenous” National Housing Strategy [19]. 

### Limitations 

Some important limitations must be taken into account in interpreting the findings. Given the relatively small program size, a total of 13 staff members participated in the study. While this represents 93% participation from the team, the sample size for the study was low. As a result, we chose not to do any sub-group comparisons within the sample. This limitation was managed through triangulating these findings with other data sources in the form of key informant interviews and client focus groups in the larger nested study. A larger sample size may have been beneficial in reaching saturation, however concept mapping methods often do not aim for generalizability, and rather focus on learnings to be considered in developing other programs. 

## 5. Conclusions

Concept mapping is a mixed methods approach that provides a structure for multiple voices to be heard and supports community engagement in developing a visual representation of community knowledge and priorities. This method allowed for articulation of what makes the program unique as an Indigenous Housing First model. Through our community-based approach, we were able to achieve strong engagement and buy-in from the participants, generating more trustworthy and reliable results that could influence program learnings. Future research may explore sampling from multiple Indigenous Housing First programs to gain both a larger sample size and for comparisons across various program features. Housing Services is a unique Indigenous housing first model that delivers holistic, client-centred supports and promotes staff wellness and team building in Hamilton, Ontario. It is through a strengths-based, culturally safe and localized approach, that Housing Services can improve health, well-being, and quality of life for Indigenous people.

## Figures and Tables

**Table 1:** Rated Statements by Cluster.

Cluster		Statement	Average Feasibility Rating	Average Importance Rating	Feasibility Rating	Importance Rating
1. Fostering Change			4.36	4.47	High	Low
Supporting clients’ goals by meeting them where they are at	1	Facilitating sobriety groups for clients	4.75	4.25	High	Low
	13	Program focuses on building and providing a circle of care for clients	4.38	4.50	High	Low
	15	Program provides support groups using a recovery framework	4.50	4.25	High	Low
	22	Working on client goals at their own pace	4.25	4.88	High	High
	23	Program provides clients and community with regular drop-ins with food, clothing, toiletries (e.g., weekly breakfast, daily life skills)	4.25	4.25	High	Low
	26	Staff de-escalate and support clients in crisis	4.13	4.13	Low	Low
	30	Staff honouring lives and gifts of clients	4.38	4.63	High	High
	53	Staff help clients to develop a toolbox with coping strategies, support, and resources	4.50	4.50	High	Low
	58	Staff supporting client self-image and growth once housed	4.13	4.88	Low	High
2. Daily Practices			4.18	4.50	Low	Low
Broad range of program activities delivered by staff	4	Staff advocating to landlords on behalf of clients (e.g., arrears concerns, eviction notices)	4.75	4.88	High	High
	7	Staff make referrals to services within DAHAC	4.25	4.75	High	High
	8	Staff providing home healthcare visits	3.75	4.63	Low	High
	14	Staff mentor clients to support them in giving back to community	3.50	4.13	Low	Low
	19	Staff accompanying clients to appointments (hospitals, social services, etc.)	3.75	4.00	Low	Low
	28	Rapid rehousing for clients that have been evicted	4.00	4.38	Low	Low
	34	Staff following through in every aspect of case plan (e.g., follow-up with referrals)	4.13	4.50	Low	Low
	38	Staff assist clients to get identification documents	4.25	4.13	High	Low
	39	Program provides external referrals (e.g., medical, addictions, 24/7 supports)	4.63	4.75	High	High
	40	Staff having monthly interactions with clients	4.25	4.88	High	High
	41	Program provides housing allotments to clients	4.00	4.63	Low	High
	42	Staff case plan with clients (i.e., assessments and safety planning)	4.38	4.63	High	High
	43	Program provides services and support in addition to housing	4.38	4.63	High	High
	46	Staff addressing food insecurity for clients (e.g., relationships with food banks, groceries)	4.38	4.38	High	Low
	49	Staff checking in through client housing transitions and on future housing needs	4.00	4.63	Low	High
	52	Program providing harm reduction supplies and supports	3.88	4.00	Low	Low
	54	Completing applications for income supports	4.38	4.25	High	Low
	55	Program hosts special events that don’t centre around alcohol/drugs (i.e., birthdays, holidays)	4.00	4.63	Low	High
	64	Program provides clients with start-up packages/welcome baskets with bedding	4.75	4.75	High	High
3. Program Uniqueness			4.14	4.55	Low	Low
Supporting specific experiences and needs of Indigenous population experiencing homelessness	2	Opening services to broader Indigenous community	3.50	4.00	Low	Low
	21	Program accepts referrals from all sources, family, community, and By-Name list	4.75	4.88	High	High
	24	Program offering different housing options (e.g., rooming house pilot)	3.63	4.25	Low	Low
	32	Providing health services and education in community settings (e.g., medical supplies, drop-in clinic)	3.88	4.38	Low	Low
	35	Staff conduct outreach activities with Indigenous homeless population sleeping outdoors	4.38	4.50	High	Low
	37	Program supports clients that are ineligible for Housing First service (healthcare connections, service referrals, material needs)	3.88	4.25	Low	Low
	45	Holistic approach to working with clients that includes mind, body, and spirit	4.50	4.75	High	High
	48	Creating a culturally safe environment for clients in every interaction and space	4.13	5.00	Low	High
	50	Re-establishing connection to Indigenous community for clients	4.13	4.88	Low	High
	61	Staff provide meals at programs	4.63	4.13	High	Low
	63	During intake, not relying only on acuity and focusing on stories	4.13	5.00	Low	High
4. Cultural Practices			4.33	4.73	High	High
Program provides access to local Indigenous practices and knowledge	3	Program provides access to traditional culture for clients (e.g., providing teachings)	4.13	5.00	Low	High
	12	Program re-establishes connection to Indigenous community for clients	4.00	4.88	Low	High
	16	Staff provide smudging in clients’ homes	4.38	4.25	High	Low
	17	Program housed within an agency that offers services from waters of life to returning to the spirit world	4.50	4.75	High	High
	51	Program housed within organization that has traditional healers	4.63	4.75	High	High
5. Partnerships and Agency Supports			3.89	4.56	Low	Low
Accountable and trusting relationships both internally and externally are essential to meeting client needs	5	Staff build and manage trusting landlord relationships	4.38	4.63	High	High
	6	Program partners with Ontario Aboriginal Housing for supportive housing	2.50	4.00	Low	Low
	11	Program ensures accountability between staff and clients and vice versa	4.25	5.00	High	High
	25	Program housed within a health access centre	4.50	3.88	High	Low
	27	Program builds in flexible funding to respond to client needs (i.e., medical supplies, for client units)	4.25	4.38	High	Low
	44	Team lead and senior management understanding and supporting all staff roles	3.88	4.88	Low	High
	56	Program provides a smooth transfer from intake to case manager	3.88	4.63	Low	High
	59	Program aware and responsive to needs of community	3.75	4.75	Low	High
	62	Program has support from all levels of DAHAC management to address bureaucratic barriers	3.75	4.88	Low	High
	65	Team lead mitigating and managing partnerships with external groups (landlords, Ontario Aboriginal Housing)	3.75	4.63	Low	High
6. Team’s Professional Skills			4.31	4.75	High	High
Program creates space for staff to self-reflect, role model and engage in self-care	9	Staff recognizing and drawing boundaries with clients	4.38	4.88	High	High
	10	Program operating from stance of hope and strengths	4.50	5.00	High	High
	18	Staff advocating for clients (i.e., shelter access, hospital care, CTOs, basic rights)	4.50	4.88	High	High
	20	Staff self-reflecting on their biases in client relationships	3.75	4.75	Low	High
	29	Managing and setting client expectations of program at intake	4.13	4.63	Low	High
	31	Empathetic staff	4.75	4.88	High	High
	33	Program consists of a supportive, close, and trusting team	4.50	4.88	High	High
	36	Program’s approach is inclusive of clients’ full identities (i.e., sexual identity/gender/culture)	4.63	4.50	High	Low
	47	Prioritizing client choice in housing search (location, housing type, roommates, etc.)	4.13	4.25	Low	Low
	57	Staff engage in self-care	3.38	4.75	Low	High
	60	Staff provides positive role modelling	4.75	4.88	High	High
